# Rearing Environment during the Endogenous Feeding Stage of *Acipenser baerii*

**DOI:** 10.3390/ani12172205

**Published:** 2022-08-26

**Authors:** Lucia Aidos, Alessandra Cafiso, Annalaura Lopez, Mauro Vasconi, Luisa M. P. Valente, Chiara Bazzocchi, Alessia Di Giancamillo

**Affiliations:** 1 Department of Biomedical Sciences for Health, University of Milan, 20133 Milan, Italy; 2 Department of Veterinary Medicine, University of Milan, 26900 Lodi, Italy; 3 Regional Health Service—Veterinary Department, ATS della Val Padana, 46100 Mantova, Italy; 4 ICBAS-Instituto de Ciências Biomédicas de Abel Salazar, University of Porto, 4050-313 Porto, Portugal; 5 CIMAR/CIIMAR—Interdisciplinary Centre of Marine and Environmental Research, Terminal de Cruzeiros do Porto de Leixões, 4450-208 Matosinhos, Portugal

**Keywords:** *Acipenser baerii*, substrate, muscle development, swimming behaviour, histometry, gene expression

## Abstract

**Simple Summary:**

In commercial hatcheries it is a common practice to rear Siberian sturgeon larvae in bare bottom tanks. Little is known about the impact of the presence of a rearing substrate during early life stages in this species. Our study aimed at comparing two different substrates (two types of Bioballs) with no substrate at all, in terms of growth, lipid metabolism, muscle development, and stress status, during the endogenous feeding stage of Siberian sturgeon. Our results suggest that the presence of a specific substrate may have positive effects in terms of growth, but it would seem important to also extend this study to the exogenous feeding stages in order to verify the mixed effect of feed and substrate on Siberian sturgeon larvae.

**Abstract:**

The aim of this study was to evaluate behaviour, growth, lipid composition, muscle development, and stress status of Siberian sturgeon larvae reared with two types of substrate: Bioballs1 (BB1) and Bioballs2 (BB2), when compared to no substrate (CTR). Sampling points were: hatching (T0), schooling (T1), and yolk-sac full absorption (T2). BB1 larvae were less active and showed no schooling behaviour. At T1 and at T2, BB1 larvae showed a significantly higher weight and total length than larvae reared in either CTR or BB2 (*p* < 0.05). The lipid content of larvae decreased over time, with little relevant differences between groups. At T2, total muscle area, slow muscle area and fast muscle area were significantly higher in larvae reared in BB1 (*p* < 0.05). No significant differences in muscle proliferation were found between groups. Real Time PCR was used for evaluating the relative expression of a pool of genes: *myod*, *myog*, *mrf4*, *igf2*, *hsp70*, *hsp90a*, *hsp90b*, and *glut2*. The expression of these genes did not seem to be much affected by the type of rearing substrate, except for *myog* and *hsp70* at T1, which was greater in BB2 larvae. Our data suggest that the presence of a substrate during this developmental period seems to have positive effects but further studies would be necessary during the exogenous feeding stage.

## 1. Introduction

The Siberian sturgeon, *Acipenser baerii*, Brandt, 1869 is the most widely farmed sturgeon and is produced mainly in China, Russian Federation, France, Spain, and Italy [1]. Nowadays, Siberian sturgeon is a threatened species [2], and is included in the International Union for Conservation of Nature Red Data List. Taking into account the continuous decrease of its natural populations due to the reduction of spawning spots, overfishing, and water pollution [3,4], it is necessary to gather as much information as possible in order to properly manage the natural populations of this species and to enhance farming practices.

During Siberian sturgeon ontogenesis there are several behavioural changes regarding rheotaxis, phototaxis, and swimming ability which are the result of both extrinsic (e.g., water temperature, current intensity, substrate typology, predation, and food resources) and intrinsic (morpho-physiological development) parameters [5]. The main factors affecting the Siberian sturgeon’s larvae seem to be the intensity of the river’s current, the type of substrate, and the food resources; for juveniles and adults, food availability plays a major role in its distribution [5].

The development and the behaviour of Siberian sturgeon from hatching until the beginning of the exogenous feeding will not be addressed as it has been already exhaustively described elsewhere [6,7]. Briefly, until 3 days post hatch (dph), healthy larvae show positive phototaxis, a preference for white substrates and vertical swimming/drift behaviour: they move upward by active movements and then, in a passive way, settle to the bottom [7]. At 4 dph, pectoral fins have developed and consequently, larvae behaviour shift from pelagic to benthic. At 7–8 dph larvae show schooling behaviour and look for covered habitats preferably with dark bottoms [7]. Schooling behaviour is characterized by the tendency to polarize, with individuals adopting the same orientation and swimming in the same direction.

Environmental enrichment is defined as an intentional increase of the degree of the environmental complexity [8], using structures such as pipes, tiles, and non-buoyant plastic strips, which are usually called “substrates” [8]. Substrates consist in functional shelters and their insertion in the environment of captive fish may provide new sensorial and motor stimulation, while decreasing stress and the occurrence of abnormal behaviors [9]. Most behavioural observations in fish have been performed in the absence of a substrate/shelter and, therefore, may not reflect the behaviour of larvae in their natural environment [10]. To date, the habitat used by early larval stages of Siberian sturgeon has not been described yet and taking into account the sensitivity of larval fish to environmental conditions [11], it seems important to evaluate the potential contribution of environmental enrichment on growth and muscle development of this species. Gravel substrates are known to be essential in the life histories of many riverine fish species, particularly in the early life stages. Indeed, in numerous studies performed with several fish species, there was an evident effect induced by the substrate presence. Peterson and Martin-Robichaud (1995) [12], tested the effect of substrate at different temperatures and found that gross yolk utilization efficiency was higher for salmon alevins reared on substrate regardless of the temperature. A positive effect of the presence of a substrate in early life stages was also found in Atlantic sturgeon (*Acipenser oxyrinchus*) [13], white sturgeon (*Acipenser transmontanus*) [14,15], and in lake sturgeon (*Acipenser fulvescens*) [16].

Effects of environmental enrichment in aquaculture, such as growth performance, behaviour, brain plasticity and neurogenesis, physiological condition, and stress-related physiological responses, have been thoroughly reviewed [9]. When subjected to environmental stimuli, the hypothalamus of fish determines the secretion of GH by the pituitary gland, which then induces the production and release of insulin growth factor (IGF) in target tissues. IGFs stimulate somatic growth by regulating cell differentiation, proliferation, and migration in tissues like bone and skeletal muscle [17]. Among the IGFs, it seems that IGF1 has a smaller effect on inducing muscle cells differentiation in rainbow trout, compared with IGF2 [18]. Therefore, *igf2* constitute a valid index of muscle growth together with other genes involved in muscle development such as *myogenin* (*myog*), *myogenic differentiation 1* (*myod 1*), and *myogenic regulatory factor 4* (*mrf4*/*herculin*/*myf6*, hereafter ‘*mrf4*’). These constitute reliable indicators to assess muscle development and growth in fish and may therefore provide information regarding growth potential [19]. Fish farming implies many operations that can be stressful for fish; environmental enrichment may have a role in helping fish handle stressful events and also improve their overall welfare [9]. Genes involved in cellular stress reactions, such as the *solute carrier family 2 member 2* (*glut2*), *heat shock protein 70* (*hsp70*)*, heat shock protein 90 alpha* (*hsp90a*), and *heat shock protein 90 beta* (*hsp90b*) are good indicators of the stress status in fish [20].

During the endogenous feeding stage, lipids contained in the yolk-sac of fish larvae represent the preferred source for growth and development, producing energy by an intense fatty acid (FA) metabolism [21]. As the energy of the yolk is limited, the more energy that goes to one process, the less there will be available for another one. Moreover, not all FA are associated with the same functions in larvae and fish metabolism with SFA and MUFA representing the main energy sources during and PUFA expressing different biological functionality for the development of the new individual [22]. For this reason, a different FA profile could be observed when different lipid metabolic rates are displayed, with SFA and MUFA catabolized more when larvae need more energy for moving.

Since little is known on the habitat preferences of the larvae of Siberian sturgeon after hatching and the impact of a substrate in these early life stages, in terms of behaviour, growth, and stress response, this study aimed to assess the environment enrichment that allows good rearing conditions without compromising larval morpho-functional aspects.

## 2. Materials and Methods

### 2.1. Experimental Set-Up

The experiment was held during April/May 2018 at the Experimental Animal Research and Application Centre of Lodi, of the University of Milan. Siberian sturgeon fertilized eggs were transported 24 h after fertilization from the “Società Agricola Naviglio” fish farm to the experimental unit. Eggs were incubated at 16 °C, then the hatching temperature was gradually increased to 19 °C: the temperature was chosen taking into account a previous experimental trial based on three rearing temperatures [23], in which a temperature of 19 °C in this phase of development produced larvae with higher muscle development potential. After hatching, larvae were randomly transferred to the 2 L experimental units (three replicates per group) until the yolk-sac was fully absorbed.

The bottom of the experimental units was covered with two different types of Bioballs which are plastic spheres usually used in biological biofilters, designed to greatly increase the surface area where bacteria can attach. Bioballs type 1 (BB1) and type 2 (BB2) differ in diameter and in shape: BB1; Φ35 mm; specific surface area 395.64 m^2^/m^3^; BB2; Φ38 mm; specific surface area 410.36 m^2^/m^3^. A group reared without additional substrate served as a control (CTR). Rearing density was of 130 larvae/1.6 L corresponding to 612 individuals/m^2^: also, in this case, fish density was chosen on the basis of a previous experimental trial [24,25], where mid-rearing density was the best compromise between zootechnical performance and economic feasibility.

Fish were reared in a recirculating aquaculture unit, composed by a sand filter, a biological submerged filter, and a UV lamp sterilization unit. Every substrate condition was tested in triplicate. Water quality parameters as oxygen, temperature, and pH were measured every day by Hach HQ 30d Portable Meter (Hach Lange, Dusseldorf, Germany); O_2_ was constantly close to the saturation value and was maintained up to the FAO suggested value (6 mg/L) described for this species during the larval stage, for all groups pH values were between 8.5 and 8.8, which fall in the physiological range described for this species in this developmental phase [26]. Measurements of ammonia, nitrite, and nitrate were monitored regularly by a Hach 2800 Portable Spectophotometer (Hach Lange, Dusseldorf, Germany) and were compliant with values recommended for Siberian sturgeon (<0.05 mg/L). Mortality was estimated by daily counting and recording of dead larvae. Eggs and larvae were exposed to an artificial photoperiod regime of 12L:12D. The experimental period matched the endogenous feeding stage of Siberian sturgeon larvae meaning that there was no need for exogenous feed, as the larvae utilized the nutrients from their yolk-sac.

### 2.2. Larval Parameters and Sampling

Sampling time points were chosen according to important steps of Siberian sturgeon larvae development: hatching (T0), beginning of the schooling phase (T1, 5 dph), and complete yolk-sac absorption phase (T2, 8 dph). Behaviour was assessed by daily counting the larvae swimming in the water column. The ratio of swimming larvae was calculated taking into account the total number of the larvae present in the rearing units. The images were taken using a digital photo camera (Nikon D750, 24 Mpixel) and were examined using an image analyse software (Optika Proview, x64, Ponteranica, Italy).

Larvae were sampled for growth assessment, lipid content, and FA profile, histological analyses, and gene expression. Larvae were collected with a beaker and euthanized by over-anaesthesia (500 mg/L) with Ethyl 3-Aminobenzoate, Methanesulfonic A (Sigma-Aldrich, Milan, Italy).

### 2.3. Lipid Content and Fatty Acid Composition

At T0, six larvae were sampled and at T1 and T2, six larvae were sampled per replicate, per treatment (*n* = 54). The extraction and determination of total lipids in larvae was performed according to the method described by Folch et al. (1957) [27], using a mixture of chloroform:methanol (2:1, *v*/*v*) and the preparation of fatty acid methyl esters (FAME) was performed according to Christie (2003) [28]. The detailed methodology may be found in a previous work [29]. 

### 2.4. Muscle Histometry

At T0, six larvae were sampled and at T1 and T2, six larvae were sampled per replicate, per treatment (*n* = 54). Larvae sampled for muscle histometry were weighed (body weight, BW), measured (total length, TL), and immediately fixed in fresh 4% para-formaldehyde in 0.01 M phosphate-buffered saline (PBS) pH 7.4 for 24 h at 4 °C; afterwards, they were dehydrated in an ordered sequence of ethanol, cleared with xylene and embedded in paraffin. From each sample three serial transverse microtome sections (5 μm-thick) were obtained and the sequential haematoxylin/eosin (HE) stain was performed to evaluate the structural aspects of the developing lateral muscle tissues and for histometry as already performed in previous studies of our group [23,25].

An Olympus BX51 light microscope provided with a DP-software program (Cell^B, Basic Imaging Software, Olympus, Italy) was used for standard histometrical techniques, in order to describe: (i) total muscle cross-sectional area (TMA), (ii) red muscle area (slow muscle cross-sectional area, SMA), and (iii) white muscle area (fast muscle cross-sectional area, FMA), (iv) lamellae fibres area (LFA), and (v) polygonal fibres area (PFA) at the three examined developmental stages: hatching (T0), schooling (T1), and yolk-sac full absorption (T2). The mean of the three serial sections was used for statistical analyses.

### 2.5. Muscle Immunohistochemistry

On other transverse sections, immunostaining was performed to detect proliferating cell nuclear antigen (PCNA). The procedure applied has been previously described in detail [30]. 

PCNA-immunopositive cells were in short described as “proliferating cells”; the relative proliferating cells number was assessed by counting the immunopositive nuclei of the muscle fibres in a tissue area corresponding to the above mentioned FMA at the three analysed developmental stages and then converted to number of proliferating cells/mm^2^.

### 2.6. RNA Extraction and cDNA Synthesis

For the molecular analyses, six larvae were sampled at T0, while six larvae were sampled per replicate, per treatment at T1 and T2 (*n* = 54). Larvae were immediately stored at −80 °C soon after the sampling procedure. Total RNA was extracted from each frozen single larval sample using the RNeasy Mini Kit^®^ (Qiagen, Hilden, Germany) following the manufacturer’s instructions with a final elution in 40 μL of RNase-free water. A double treatment with DNase enzyme (Qiagen, Hilden, Germany) was performed in order to remove any genomic DNA contamination, according to the manufacturer’s instructions. The quantification and purity of the extracted RNA was assessed using the Nanodrop ND-1000 Spectrophotometer (Thermo Fisher Scientific, Waltham, MA, USA). Five hundred nanograms of RNA was retro-transcribed to cDNA using the Quantitect Reverse Transcription Kit^®^ (Qiagen, Hilden, Germany) following the manufacturer’s protocol. An additional reaction without retrotranscriptase enzyme was performed to verify the complete DNA removal. The cDNAs were stored at −80 °C until subsequent use.

### 2.7. Gene Identification and Primer Design

The following genes were selected for a differential expression analysis according to previous studies performed on Siberian sturgeon [24,31]: (i) *glut2*, *hsp70*, *hsp90a*, and *hsp90b* genes involved in cellular stress reactions; (ii) *myod*, *myog*, and *mrf4* genes involved in muscle development, and *igf2*, related with muscle growth. Additionally, *rpl6* (coding for *ribosomal protein L6*) and *gapdh* (coding for *glyceraldehyde 3-phosphate dehydrogenase*) genes were used as references as described in Aidos et al. (2020a, 2020b) [24,31]. The molecular analyses were performed following the minimum information for publication of quantitative real-time PCR experiments (MIQE) guidelines [32]. Primer sequences used for the amplification of *glut2*, *hsp70*, *hsp90a* and *hsp90b*, *myog*, *igf2*, *rpl6*, and *gapdh* gene fragments were previously described in Aidos et al. (2020a) [24]. Specific primers for the amplification of *myod* (F: 5′-AGACCACCAATGCTGACCG-3′; R: 5′- GTCTCTGGTTGGGGTTTGT-3′; amplification size 120 bp) and *mrf4* (F: 5′-TTGCAGGGAAATGTGGAC-3′; R: 5′-GCTAAACTGGAATGATCGGA-3′; amplification size 125 bp) gene fragments were de novo designed and validated as described previously in Aidos et al. (2020a) [24].

### 2.8. Gene Expression Profiles

The expression of genes coding for muscle development/growth and stress was analyzed by quantitative PCR (qPCR) in larvae collected at the three rearing conditions (CTR, BB1 and BB2). 

The cDNA samples were used as a template in qPCR using a BioRad iQ5 Real-Time PCR instrument (Bio-Rad, Hercules, CA, USA) and Universal SYBR^®^ Green Supermix (Bio-Rad, Hercules, CA, USA) as a fluorescent molecule. The qPCR mixture included 1 µL of cDNA per reaction and the final concentration of forward and reverse primers was 150 nM for each amplified gene; qPCR reactions were performed in duplicate. The thermal profile for the amplification of *myod* and *mrf4* genes was 98 °C for 30 s, 40 cycles of 98 °C for 15 s, 58 °C for 30 s, and a melting profile included after the last amplification cycle. PCR conditions for *myog*, *hsp70*, *hsp90a*, *hsp90b*, *rpl6*, and *gapdh genes* and *igf2 and glut2* genes respectively have been reported previously elsewhere [24,31].

Cycle threshold (Ct) values were determined for each gene. Results from the iQ5 Software Data Analysis module (Bio-Rad, Hercules, CA, USA) were imported into Microsoft Excel and transformed to relative quantities using the comparative Ct method and specific efficiencies for each amplified gene [33]. The expression of each gene related to BB1 and BB2, for both T1 and T2, was compared to the calibrator sample CTR, and the relative expression values were calculated after a ΔΔCt measure using the *rpl6* and *gapdh* genes as references. The amplified *myod* and *mrf4* gene fragments were loaded on agarose gels, purified and sequenced and the obtained sequences were deposited in GenBank (https://www.ncbi.nlm.nih.gov/genbank/ (accessed on 21 January 2022).

### 2.9. Statistical Analysis

Statistical analysis was performed with SAS statistical software (version 9.3, Cary Inc., Cary, NC, USA). Data from larval zootechnical parametrs, lipid content/FA composition, the histometrical analyses (TMA, SMA, FMA, LFA and PFA), PCNA cellular counts, and molecular analyses were analysed using 2-way ANOVA with substrates (CTR, BB1, and BB2) and developmental stages (T0, T1 and T2) as main factors, and co-variated for the total area corresponding to the TMA. All data were tested for normality using a Shapiro–Wilk test and for homogeneity of variance using a Cochran test. The data are presented as least-square means (SEM). Differences between means were considered significant at *p* < 0.05.

## 3. Results

### 3.1. Development, Behaviour, Survival and Growth

Once released in the units without any substrate (CTR group), newly-hatched larvae exhibited swim-up and drift behaviour, swimming upwards in the water column and falling down to the bottom in a continuous movement. Larvae released in the BB1 or BB2 units showed the same behaviour as the CTR group but only for a limited amount of time until the substrate was encountered. At this point, most of the larvae from these groups remained in the bottom, mainly the ones from group BB1. Few larvae from groups BB1 and BB2 showed schooling behaviour while larvae from the CTR group all swam in schools at 5 dph. After schooling, larvae from the CTR group remained mostly in the bottom until the yolk-sac was fully absorbed. On the opposite, larvae from groups with a substrate (BB1 or BB2) kept on swimming in the water column from 4–5 dph until the yolk-sac was fully absorbed.

**Figure 1:**
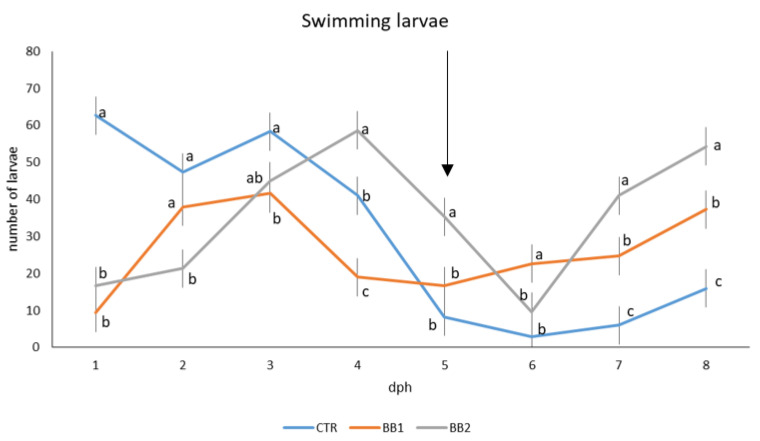
Number of larvae of *A. baerii* swimming in the water column for each treatment, from T0 to T2. Blue line, CTR, orange line, BB1, grey line, and BB2. Error bars indicate the standard error of the mean for each treatment/stage of development; ^a,b,c^ Means with different superscripts differ significantly between treatments (*p* < 0.05); arrow indicating schooling phase (T1).

Figure 1 shows the records of the larvae that swam in the water column from hatching until the end of the trial. Until 3 dph, the number of larvae swimming in the water column was significantly higher in group CTR. From day 4–5 post-hatch the situation started to invert: the number of larvae swimming in the water column was significantly higher in the BB2 group. The number of swimming larvae from group BB1 was in between the other two groups throughout the trial.

**Figure 2:**
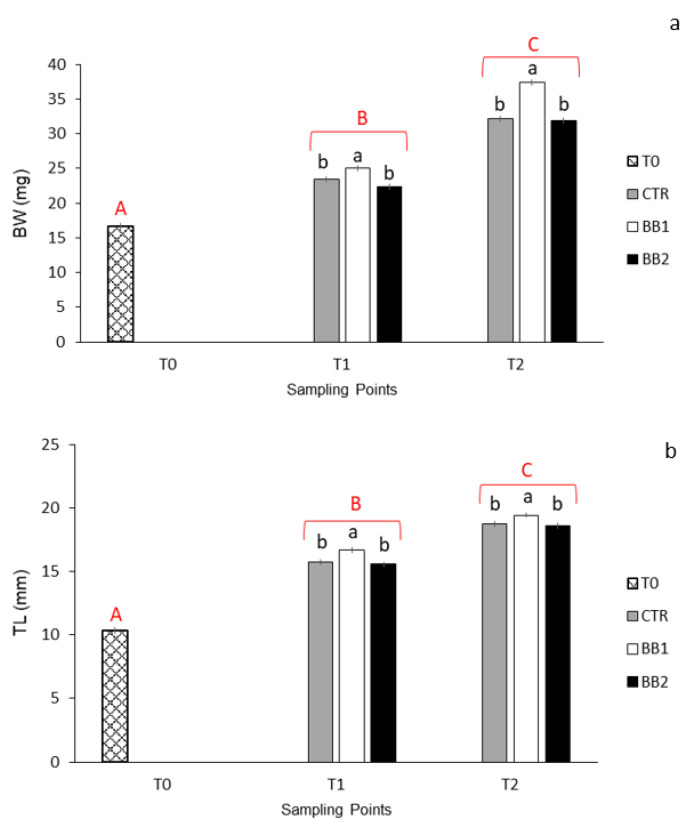
(**a**) Larval growth expressed in mg of BW; (**b**) Larval length expressed in mm of TL; error bars indicate the standard error of the mean for each treatment/stage of development; ^a,b^ Means with different superscripts differ significantly between treatments (black font) or stages of development (red font) (*p* < 0.05); ^A,B,C^ Means with different superscripts differ significantly between treatments (black font) or stages of development (red font) (*p* < 0.01).

Survival rates were high, between 98.2 and 99.0% and no significant differences were found between the groups. BW significantly increased from one stage of development to the other, irrespective of the substrate (Figure 2a; *p* < 0.01). At T1, larvae reared with substrate BB1 showed a significantly higher weight than larvae from the other two groups and this difference became bigger at T2 (Figure 2a; *p* < 0.05).

TL significantly increased from one stage of development to the other, irrespective of the type of substrate (Figure 2b; *p* < 0.01). Both at T1 and T2, larvae reared in the substrate BB1 were longer than larvae from groups CTR and BB2 (Figure 2b; *p* < 0.05). The interaction between developmental stages and substrate was not significant neither for BW nor for TL.

### 3.2. Lipid Content and Fatty Acid Composition

Our results show that the stage of development (T0 vs. T1 vs. T2) has a stronger influence on lipid content of Siberian sturgeon larvae than the experimental group (CTR, BB1 and BB2). Total lipid content decreased between T1 and T2 (*p* < 0.001, Table 1), without significant differences among experimental groups (Table 1). 

Differences were also found in the FA profile. Considering the stage of development (Table 1), the group of saturated FA (SFA) increased between T0 and T2 (*p* < 0.001), whilst the group of monounsaturated FA (MUFA) decreased (*p* < 0.001). The group of polyunsaturated FA (PUFA) showed a significant decrease (*p* < 0.05) only in T0 and T1. Two FA in this group, namely arachidonic acid (ARA, 20:4 n-6) and docosahexanoic acid (DHA, 22:6 n-3), had an opposite trend increasing between T0 and T2 (*p* < 0.01). This trend was also observed in their ratios calculated above eicosapentaenoic acid (EPA, 20:5 n-3), namely DHA/EPA ratio and ARA/EPA ratio. 

Considering the type of substrate tested (Table 1), larvae reared on substrate BB2 did not show great differences with the CTR group, with the exception of 14:0, found higher in BB2 than in CTR at T2 (*p* < 0.001). Larvae reared on substrate BB1 showed significant differences with both CTR and BB2 groups at T1; particularly, BB1 had higher amounts of 18:1 n-7, DHA and, DHA/EPA ratio and lower amounts of linoleic acid (LA, 18:2 n-6). At T2, BB1 larvae showed higher amounts of 20:2 n-6 than CTR and BB2 larvae.

Finally, significant differences were observed between BB1 and BB2 groups at T1, mainly alpha-linolenic acid (ALA, 18:3 n-3) was higher in BB1 and 20:3 n-6 and ARA were higher in BB2 larvae. At T2, BB1 larvae showed higher levels of stearic acid (18:0) and lower amounts of palmitoleic acid, total MUFA and 18:3 n-6 than BB2 larvae.

### 3.3. Histological and Histometrical Analyses

**Figure 3:**
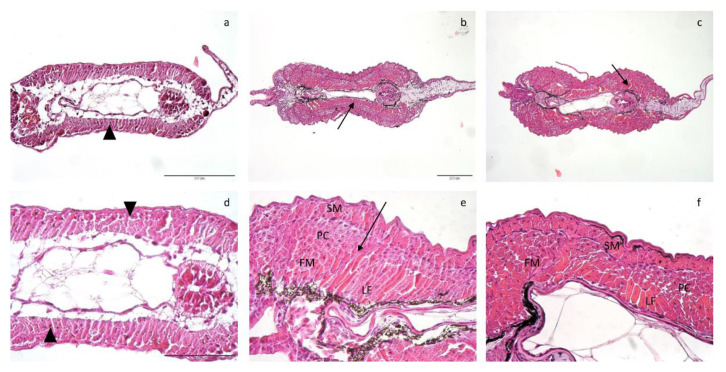
HE staining representative figures for: (**a**,**d**) hatching; (**b**,**e**) schooling; and (**c**,**f**) yolk-sac full absorption, at BB1. FM, fast muscle; SM, slow muscle; monolayer, arrowheads; multilayers, arrows; LF, lamella-shaped fibre; PC, polygonal cells. (**a**) has the scale bas as indicated in the figure itself: 200 µm; (**c**) has the same scale bar as located in (**b**): 200 µm. (**e**,**f**) have the same scale bas as indicated in (**d**): 100 µm.

Histological analyses revealed an anatomically regular muscle development: at hatching larvae presented an outer monolayer of slow muscle cells (SM) and an inner monolayer of fast muscle cells (FM). From T0 to T2 there was an expansion of both layers (SM and FM), from monolayer to multilayers (arrowheads and arrows, respectively; Figure 3a–f).

**Figure 4:**
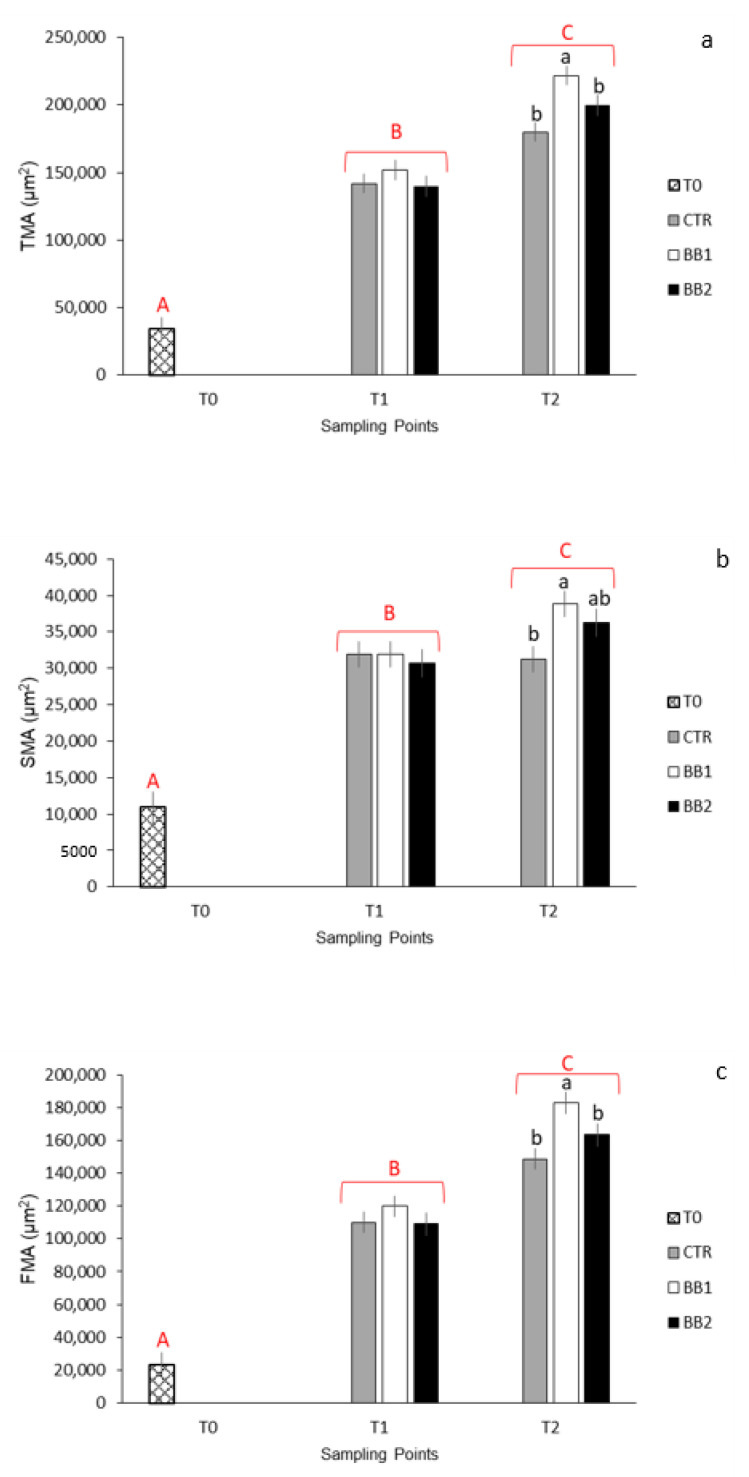
Quantitative representation of: (**a**) total muscle cross-sectional area (TMA): area expressed in µm^2^; *n* = 18/group; (**b**) red muscle area (slow muscle cross-sectional area, SMA), area expressed in µm^2^; *n* = 18/group; (**c**) white muscle area (fast muscle cross-sectional area, FMA), area expressed in µm^2^; *n* = 18/group; ^a,b^ Means with different superscripts differ significantly between treatments (black font) or stages of development (red font) (*p* < 0.05); ^A,B,C^ Means with different superscripts differ significantly between treatments (black font) or stages of development (red font) (*p* < 0.01).

Histometrical results are presented in Figure 4a–c. TMA, SMA, and FMA significantly increased from one stage of development to the other (Figure 4a–c; *p* < 0.001). At the schooling stage, there were no significant differences regarding TMA; at the end of the trial, though, TMA was significantly higher for larvae reared in substrate BB1 (Figure 4a; *p* < 0.05). Similarly, there were no significant differences at T1 for SMA but, at T2, SMA was significantly higher for larvae reared in substrate BB1 when compared to larvae reared without any substrate (CTR) (Figure 4b; *p* < 0.05). At the schooling stage there were no differences between groups concerning the FMA. At T2, FMA was significantly higher for larvae reared with substrate BB1 (Figure 4c; *p* < 0.05). The interaction between developmental stages and substrate was not significant for TMA, SMA, or FMA.

**Figure 5:**
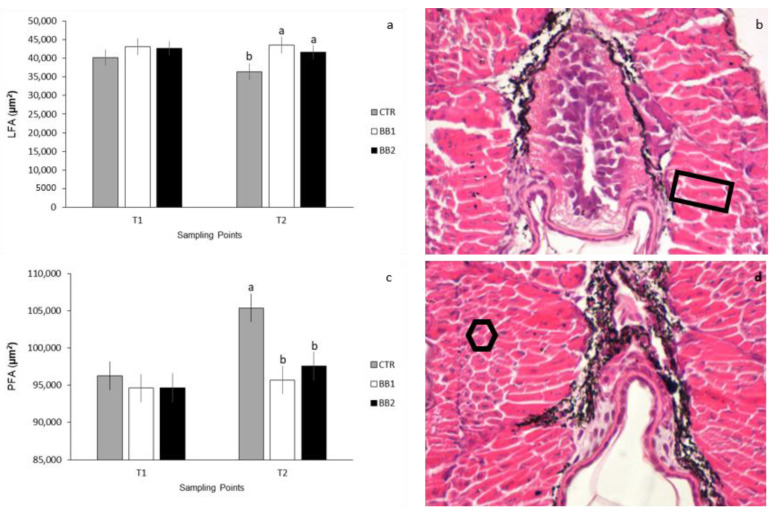
(**a**) Quantitative representation of lamella-shaped fibres area, LFA; (**b**) HE staining representative figures for yolk-sac full absorption, at BB1; rectangle indicating lamella-shaped fibre; (**c**) quantitative representation of and polygonal cells area, PFA. Area expressed in µm^2^; *n* = 18/group; ^a,b^ Means with different superscripts differ significantly between treatments (*p* < 0.05); (**d**) HE staining representative figures for yolk-sac full absorption, at BB2; hexagon indicating polygonal cell.

In the FMA at T1 and T2 two different types of cells were identified: lamella-shaped fibres (LF) that occupy an inner position, and polygonal cells (PC) which are located externally (Figure 5). As for the areas occupied by LF and PC, no differences were found between groups at the schooling stage (Figure 5a,b respectively). At the yolk-sac absorption stage, however, the area occupied by the lamellae-shaped fibres was significantly higher in larvae reared in substrates BB1 and BB2 rather than larvae reared without any substrate (CTR) (Figure 5a; *p* < 0.05). The area occupied by the polygonal cells was significantly higher for larvae reared without any substrate (CTR) than in larvae from group BB1 or BB2 (Figure 5b; *p* < 0.05,). The interaction between developmental stages and substrate was not significant for LF or PC, too.

### 3.4. Immunohistochemistry and Cell Counts

**Figure 6:**
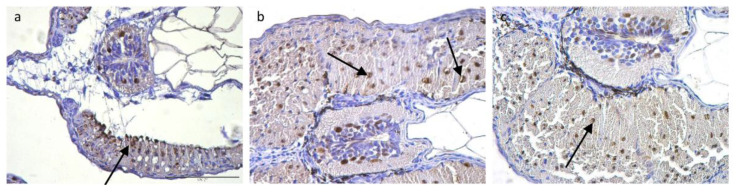
Representative images of PCNA-immunolocalization (arrows) at different timepoints: (**a**) T0; (**b**) T1, BB1; (**c**) T2, CTR. Scale bar: 100 µm.

Anti-PCNA immunostaining of the lateral muscle was observed in FM nuclei (Figure 6a–c, arrows). At T0 proliferation of the FM fibres was significantly higher (4.00 PCNA/mm^2^) than at T1 (1.13 PCNA/mm^2^) and T2 (0.88 PCNA/mm^2^) (*p* < 0.001), regardless of the type pf substrate. Both at T1 (CTR 0.19 PCNA/mm^2^; BB1 0.86 PCNA/mm^2^; BB2 1.21 PCNA/mm^2^) and at T2 (CTR 0.96 PCNA/mm^2^; BB1 0.82 PCNA/mm^2^; BB2 0.87 PCNA/mm^2^) there were no significant differences between groups (*p* > 0.13). The interaction between developmental stages and substrate was not significant.

### 3.5. Environmental Stress, Muscle Development, and Growth-Related Gene Expression

**Figure 7:**
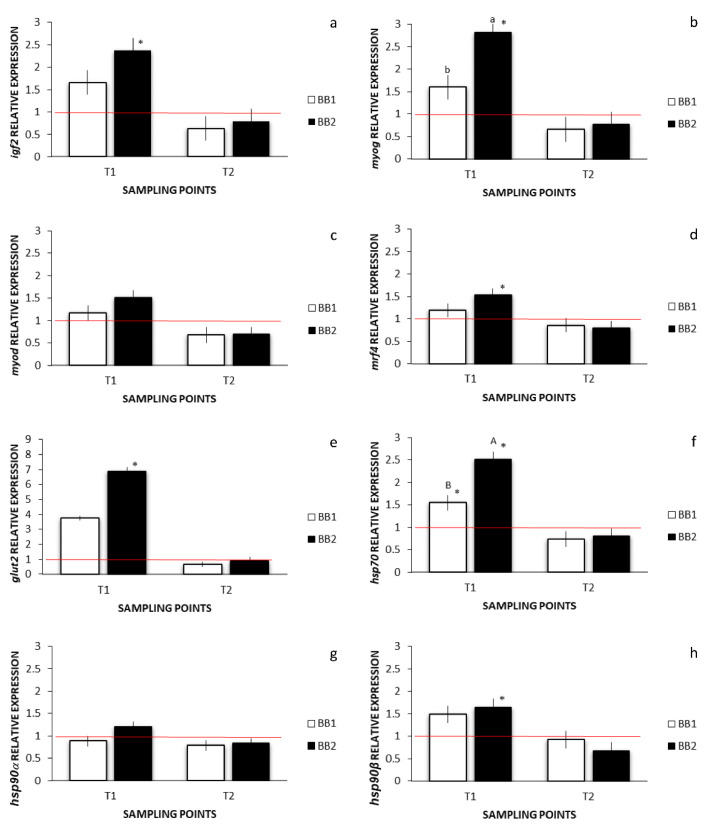
Relative gene expression of: (**a**) igf2; (**b**) *myog*; (**c**) *myod*; (**d**) *mrf4*; (**e**) *glut2*; (**f**) *hsp70*; (**g**) *hsp90a*; (**h**) *hsp90b*; ^a,b^ Means with different superscripts indicate significant differences of at least *p* < 0.05, between BB1 and BB2; ^A,B^ Means with different superscripts indicate significant differences of at least *p* < 0.01, between BB1 and BB2; asterisks indicate a significant difference between BB1 or BB2 and CTR, corresponding to the red line, which indicates the up- and down-regulation limit at least at *p* < 0.05.

The specificity of primers designed for the amplification of *myod* and *mrf4* gene fragments of Siberian sturgeon was assessed by Sanger sequencing. The obtained sequences were deposited in GenBank under the accession numbers OM326825 and OM326824, respectively. The relative expression of the muscle development/growth-related genes and of stress-related geneses at T1 and T2 is shown in Figure 7.

At T1 genes *myog* and *hsp70* showed a higher expression in larvae from the BB2 group compared to BB1 (Figure 7b,f; *p* < 0.05 and *p* < 0.01, respectively). At T2 no significant differences were found in the expression between groups for all genes analysed.

The expression of all genes was up-regulated at all groups at T1 when compared to the CTR group (expressed in Figure 7 as the red line indicating the up- and down-regulation boundary) except for the gene *hsp90a* for the BB1 larvae. However, this upregulation was not always significant. Precisely, the expression of the genes *igf2*, *myog*, *mrf4*, *glut2*, *hsp70*, and *hsp90b* at T1 for the BB2 group was significantly up-regulated when compared to the CTR group (Figure 7a–h; *p* < 0.05). Larvae from the BB1 group showed a significant up-regulation only for the gene *hsp70* (Figure 7f; *p* < 0.05). Larvae from the BB1 group showed, however, a trend towards statistical significance, when considering the up-regulation of the *glut2* gene compared to CTR group at T1 (Figure 7e; *p* = 0.0647). Always at T1, the *hsp90a* gene was more expressed in larvae from the BB2 group in respect to the BB1, but there was no significance, even if there was a tendency towards significance (Figure 7g; *p* = 0.0639). At T2, instead, all genes at both BB1 and BB2 were not significantly down-regulated (Figure 7).

## 4. Discussion

The present work investigated two rearing substrates (BB1 or BB2) plus a control group without any substrate (CTR) during the endogenous feeding period of Siberian sturgeon larval phase. Both the behaviour observations and the morpho-functional data confirmed performance differences among the substrate groups. 

We found that, in the presence of a substrate, Siberian sturgeon larvae during the first 5 dph preferred to hide in the interstitial spaces of the Bioballs rather than swimming in the water column. On the contrary, in the same period, larvae reared with no substrate were always swimming in the water column. This situation from the schooling stage onward was inverted when larvae left the Bioballs and started to swim in the water column. These results are in agreement with a study on the impact of different substrates on the larvae behaviour of Atlantic sturgeon: until day 6 post-hatch, a large majority of the larvae preferred to hide in the middle of the gravel substrate than standing on the sand where there were no hiding spots whereas, from this day onward, the majority of the larvae started to actively swim in the water column [13]. Also McAdam (2011) [10], found that white sturgeon yolk-sac larvae hid when exposed to porous substrates and nearly all larvae drifted in response to nonporous substrates. It appears that in early life stages of sturgeon larvae seek for shelter. On the contrary, Gisbert et al. (1999) [34], found that Siberian sturgeon during pre-larval development were positively phototactic and preferred white bottoms and did not show any preference for bottom concealment, and this fact can probably be explained by the different substrate employed. Recently, the swimming behaviour of Siberian sturgeon larvae has been assessed in relation to the fish feeding habits [35], but the effect of a substrate was not taken into account and therefore it is not possible to compare with the results obtained in the present study.

Gene expression gives interesting inputs regarding the development stages: at the end of the full absorption of the yolk-sac (T2) there were no differences in the expression of the investigated genes either between the two types of substrates or between these and the control group. At the schooling stage, however, differences have been observed in the gene expression, suggesting that the schooling stage (T1) is a particular step of development in this species, where larvae are more sensitive to environmental stimuli, as already observed by our group in previous studies with this species [23,24,25,31].

In our study, we found differences both in weight and in length among substrates. Type 1 Bioballs produced heavier and longer larvae than the other two groups. Larvae reared with substrate BB2 showed a higher expression of *myog*, a gene involved in the muscle differentiation process [36], when compared to BB1, and higher expression of *igf2* and *mrf4* when compared to the CTR. These results may indicate a greater growth potential of larvae reared in BB2, which can perhaps be observed in the exogenous feeding phase. Contrary to mammals, IGF2 protein seems indeed to exert greater effects on inducing differentiation in rainbow trout muscle cells compared to IGF2 [18]. In gilthead sea bream (*Sparus aurata*), it was found that IGF2 has stronger effects on myocyte proliferation and is more potent in stimulating muscle growth than IGF1 [18]. Wood et al. (2005) [37], observed that IGF2 is expressed at earlier stages and at higher levels than IGF1 in fish, which was confirmed by Patruno et al. (2008) [38], in European seabass *Dicentrarchus labrax*.

There are several studies on the impact of environmental enrichment on larval and juvenile performance and physiology of various sturgeon species. Gessner et al. (2009) [13], studied the effect of two different substrates (sand and gravel) in Atlantic sturgeon larvae and found significant differences in terms of total length and wet mass. White sturgeon larvae reared on unenriched substrates tended to grow more slowly, showed a lower condition factor, and also exhibited a delayed gut development and a reduced rate of yolk-sac absorption (at 15 dph) compared with those reared with enriched substrates [14]. Bates et al. (2014) [15], observed increased growth and reduced stress hormone levels in white sturgeon yolk-sac larvae (YSL) reared in substrate. Still in white sturgeon, the joint effect of temperature and substrate (gravel) was studied and it seems that these factors affect size; yolk absorption efficiency was independent of temperature but was significantly higher in gravel-reared larvae; survival was higher in YSL reared in gravel [39]. In another study with white sturgeon, larvae reared in gravel and artificial substrate were larger than those reared without substrate; additionally, gravel-reared larvae had higher whole-body glycogen concentrations relative to bare-tank-reared larvae [40].

During the endogenous feeding stage, lipids contained in the yolk-sac of fish larvae represent the preferred substrate for growth and development, producing energy by an intense FA metabolism [21]. Total lipid content of larvae analysed in this study decreased between T1 and T2, indicating a depletion of lipid body reserves of starving larvae when the yolk-sac is completely absorbed. As the energy of the yolk is limited, the more energy goes to one process, the less will be available for another one. Since larvae move less when settled in a substrate, more energy from the yolk is available for growth, even if the lipid content of larvae analysed in this trial was not different among groups. These data suggest that sturgeon larvae from all the groups used energy to promote growth at the same extent, independently of the substrate tested.

Generally, not all the FAs are metabolized at the same rate for growth during fish larvae early development, with SFA and MUFA providing for the most of the energy needed [21]. Interestingly, we observed that the MUFA decreased during the experimental time, suggesting a deeper catabolic activity toward this class of FA in order to produce energy. This is in agreement with the knowledge regarding the pivotal role of MUFA as major energetic fuel during embryonic and larvae development in sturgeon, that is accompanied by a contemporary storage of PUFA to meet the requirement of growth and survival [41]. In this study, PUFA content did not show a significant difference between T0 and T2. This is particularly important because PUFA, especially ARA, EPA, and DHA have been demonstrated to possess a primary biological function for fish larvae development in several species, fundamental for survival, normal growth, and development of larvae [42,43,44]. Accordingly, the levels of ARA and DHA showed an increment between T0 and T2, indicating an enrichment of these FA in the yolk-sac of sturgeon embryos and a preservation from depletion during the earliest larval stage. 

Furthermore, Ishizaki et al. (2001) [45], suggested that certain PUFA (mainly DHA) could influence specific behavioural traits including the schooling performance in early fish larvae. Particularly, the authors demonstrated that yellowtail (*Seriola quinqueradiata*) larvae that were fed a high content of DHA schooled earlier. In our trial, sturgeon larvae were in starvation conditions so availability of FA was only dependent on the inner yolk-sac reserves. In a study with herring *Clupea harengus* (L.) it was observed that swimming larvae contain more DHA in their body compared with individuals with abnormal swimming behaviour [46]. Based on these findings, we would have expected a lower content of DHA in BB1 larvae which showed the least swimming behaviour at the schooling phase. On the contrary, the highest levels of DHA at T1 were observed in BB1 rather than in BB2 and CTR larvae. BB1 larvae also showed the highest ARA content and, consequently, the highest ratios of DHA/EPA and ARA/EPA. In literature, it is indicated that EPA competes with both ARA and DHA in the growth metabolism of many fish species, influencing the biological activity of the metabolites produced. DHA/EPA ratio is considered fundamental for a correct embryonic development as well as in the larval stage of Siberian sturgeon [41]. A DHA/EPA ratio equal or greater than two has been indicated as adequate in cold and temperate water fish species [47].

In this study, we found values for DHA/EPA ranging from 5.35 to 5.66, indicating optimal FA pattern of larvae regardless of the presence or the typology of substrate tested. At T2, the most interesting difference was found for the MUFA content which was lower in BB1 larvae. Since this group comprised the heaviest and longest animals these results suggest that even at higher growth rates, the metabolism of sturgeon larvae operates a selective depletion of this class of FA and preferentially retains PUFA which is biologically fundamental for the correct development of fish.

Another fundamental aspect for fish development is muscle growth which is the result of hypertrophy and hyperplasia in teleosts. Hypertrophy takes place during post-embryonic life, when it reaches a functional maximum [48], and has been described for several species such as carp (*Cyprinus carpio* L.) [49], cod [50], salmon [51], and in several other marine species as reviewed by Valente et al. (2013) [19]. A considerable difference between sturgeons and teleosts is that the latter present the white muscle entirely made up of cylindrical cells from hatching to the adult life. Instead, in sturgeons, white muscle is initially composed of multinucleated muscle lamellae which, later on, give place to polygonal cells [52], in a process that is not yet fully clear. In this study it was possible to observe in all groups a profound change in the three timepoints considered: from hatching through schooling until the full yolk-sac absorption stage, fast fibres undergo a phenomenon of hypertrophy/hyperplasia, increasing their number and size. There were no qualitative nor morphological differences among the three tested substrates regarding the stages of development. A broadly used method for measuring muscle growth consists of calculating the cross-sectional muscle areas that provide an indicator of hypertrophic or hyperplastic growth [48]. Larvae reared in the BB1 substrate showed higher cross-sectional areas of total muscle (TMA), slow-muscle (SMA), and fast-muscle (FMA), which is in agreement with the fact that these larvae were also significantly heavier and longer than larvae belonging to group CTR or BB2.

Differences regarding muscle development have been noticed in the ultimate polygonal cells and in the primary lamellae-shaped fibres: larvae reared in bare units had more definitive polygonal cells than larvae from groups BB1 or BB2. In the latter, instead, there were more lamellae-shaped fibres. It appears that an increase in the conversion/differentiation of the primitive lamellae in definitive polygonal cells is related to the reduction in both length and weight of the CTR group. These preliminary results give an indication that larvae reared with a substrate are similar in terms of muscle development, while in the group of larvae raised with no substrate at all, it would seem that there is an acceleration of muscle development in its final form.

The rate of replication of the fast muscle cells was assessed, according to Rowlerson and Veggetti (2001) [48]. PCNA counts revealed no significant differences between groups, both at T1 and at T2, suggesting that the higher weight of the BB1 group is not associated with hyperplastic growth, but rather with the hypertrophy of the existing FM cells. The fact that in the BB1 group larvae were less active may justify the higher growth in terms of BW and TL but the reason behind it is not clear, also taking into account that no significant differences were found in the FA profile and content between groups. Further research would be necessary to clarify this issue.

As for the genes involved with the stress response, larvae reared with BB2 showed a significant expression or a tendency to be expressed. There are no studies regarding molecular analyses on sturgeons, but there are studies in other fish species that indicate a positive effect of environmental enrichment on welfare in terms of stress reduction. In South American catfish *Rhamdia quelen* (*Heptapteridae*) the presence of a shelter has been shown to reduce stress (as indicated by plasma concentrations of cortisol) [9]. In a study with juvenile black rockfish *Sebastes schlegelii*, the enrichment type and amount had significant effects on the basal stress level, measured as cortisol level and opercular beat rate: the authors found that the optimal basal stress level for fish was achieved by providing a medium-amount (approximately 50% floor space coverage) of mixed enrichment [53]. Two contrasting rearing environments (barren and structurally enriched) in postlarvae of grass carp (*Ctenopharyngodon idella*) up to fingerling stage [54]. The structurally complex substrate reduced the stress level as indicated by the lower expression of stress related genes. Larvae reared in BB2 show a significant higher expression of the gene *hsp70*, but only in the schooling phase, indicating that this substrate is not the most suitable one in this phase of development. The results are in accordance with a previous study where a freshwater cichlid, the *Pelvicachromis pulcher*, revealed high levels of *hsp70*, thus indicating that the quartz substrate represents a source of stress for the larvae of this species, which try to recover through an over-expression of *hsp70* [55].

## 5. Conclusions

Rearing environment during early stages of development can influence the muscle growth potential, animal well-being, and the quality of the final flesh [56]. For these reasons, it is important to optimize the larval rearing conditions to maximize posterior growth performance. Considering this fact and according to our results, providing a substrate with characteristics similar to those of BB1 seems to give a positive impact on Siberian sturgeon in early phases of development.

The identification of age-specific effects of the substrate condition on survival rate, muscular morphogenesis, and stress parameters suggests that the presence of the substrate may contribute to sturgeon growth in early aging phases. Future studies involving exogenous feeding could expand knowledge on the effects of substrate not only for hatchery practices, but particularly for conservation aquaculture, and for habitat restoration to enhance natural propagation.

## Figures and Tables

**Table 1:** Lipid content (mg/larvae) and fatty acid composition (g 100 g^−1^ of total fatty acids) of larvae according to the stage of development (T0, T1, T2) and the different treatments tested (CTR, BB1, BB2). Data are presented as LSM ± SEM.

	T0	T1					T2					
		CTR	BB1	BB2	Sign	T1 Mean	CTR	BB1	BB2	Sign	T2 Mean	Sign T
**Total lipid**	3.36 ± 0.05 ^A^	3.01 ± 0.16	3.05 ± 0.29	2.81 ± 0.27	ns	2.96 ± 0.14A	2.29 ± 0.52	2.23 ± 0.33	2.36 ± 0.22	ns	2.29 ± 0.08 ^B^	<0.001
**Fatty acid profile *(g 100 g*^−1^ *of total fatty acids)***												
**14:0**	0.84 ± 0.06 ^A^	0.86 ± 0.04	0.80 ± 0.01	0.81 ± 0.01	ns	0.83 ± 0.02 ^A^	0.69 ± 0.00 ^b^	0.73 ± 0.12 ^a^	0.74 ± 0.01 ^a^	<0.001	0.72 ± 0.01 ^B^	<0.001
**16:0**	19.45 ± 0.07 ^C^	19.96 ± 0.12	19.84 ± 0.07	19.94 ± 0.07	ns	19.91 ± 0.05 ^B^	20.29 ± 0.06	20.54 ± 0.05	20.49 ± 0.17	ns	20.44 ± 0.06 ^A^	<0.001
**18:0**	4.00 ± 0.02 ^C^	4.71 ± 0.08	4.70 ± 0.06	4.79 ± 0.08/	ns	4.73 ± 0.17^B^	5.65 ± 0.07 ^a,b^	5.80 ± 0.06 ^a^	5.50 ± 0.06 ^b^	<0.05	5.65 ± 0.19 ^A^	
**SFA**	24.29 ± 0.11 ^C^	25.53 ± 0.24	25.34 ± 0.11	25.54 ± 0.14	ns	25.47 ± 0.10 ^B^	26.63 ± 0.11	27.07 ± 0.09	26.73 ± 0.20	ns	26.81 ± 0.09 ^A^	<0.001
**16:1 n-7**	3.00 ± 0.02 ^A^	2.85 ± 0.01 ^a,b^	2.81 ± 0.02 ^b^	2.87 ± 0.01 ^a^	<0.05	2.84 ± 0.01 ^B^	2.61 ± 0.02 ^a,b^	2.57 ± 0.02^b^	2.66 ± 0.02 ^a^	<0.01	2.61 ± 0.01 ^C^	<0.001
**18:1 n-9**	34.63 ± 0.10 ^A^	34.01 ± 0.12	33.95 ± 0.10	33.97 ± 0.08	ns	33.97 ± 0.06 ^B^	32.89 ± 0.10	32.57 ± 0.10	32.94 ± 0.13	ns	32.80 ± 0.07 ^C^	<0.001
**18:1 n-7**	2.65 ± 0.01 ^B^	2.66 ± 0.01 ^b^	2.71 ± 0.01 ^a^	2.67 ± 0.00 ^b^	<0.001	2.68 ± 0.01	2.76 ± 0.01	2.78 ± 0.01	2.79 ± 0.01	ns	2.78 ± 0.01 ^A^	<0.001
**20:1 n-9**	1.11 ± 0.05 ^A^	1.08 ± 0.01	1.06 ± 0.01	1.06 ± 0.01	ns	1.07 ± 0.01 ^B^	1.01 ± 0.01	0.97 ± 0.02	1.01 ± 0.01	ns	1.00 ± 0.01 ^C^	
**MUFA**	41.39 ± 0.10 ^A^	40.59 ± 0.14	40.52 ± 0.13	40.57 ± 0.09	ns	40.56 ± 0.07 ^B^	39.27 ± 0.11 ^a,b^	38.89 ± 0.11 ^b^	39.40 ± 0.15 ^a^	<0.05	39.19 ± 0.09 ^C^	
**18:2 n-6**	12.01 ± 0.05 ^A^	11.39 ± 0.05 ^a^	11.26 ± 0.02 ^b^	11.41 ± 0.03 ^a^	<0.05	11.35 ± 0.02 ^B^	10.77 ± 0.05 ^a,b^	10.64 ± 0.06 ^b^	10.86 ± 0.02 ^a^	<0.01	10.76 ± 0.03 ^C^	<0.001
**18:3 n-6**	2.13 ± 0.01 ^A^	2.06 ± 0.01	1.99 ± 0.03	2.05 ± 0.01	<0.05	2.03 ± 0.01 ^B^	1.89 ± 0.01 ^a,b^	1.86 ± 0.01 ^b^	1.91 ± 0.02 ^a^	<0.05	1.89 ± 0.01 ^C^	<0.001
**18:3 n-3**	1.75 ± 0.08 ^A^	1.62 ± 0.02 ^a,b^	1.59 ± 0.01 ^b^	1.63 ± 0.01 ^a^	<0.05	1.61 ± 0.01 ^B^	1.46 ± 0.01	1.43 ± 0.01	1.48 ± 0.01	ns	1.45 ± 0.01 ^C^	<0.001
**20:2 n-6**	0.54 ± 0.01 ^B^	0.54 ± 0.00 ^b^	0.56 ± 0.00 ^a^	0.54 ± 0.00 ^a,b^	<0.05	0.50 ± 0.00 ^B^	0.56 ± 0.01 ^b^	0.58 ± 0.00 ^a^	0.56 ± 0.00 ^b^	<0.01	0.57 ± 0.00 ^A^	<0.001
**20:3 n-6**	0.64 ± 0.01 ^A,B^	0.60 ± 0.01 ^a,b^	0.61 ± 0.00 ^a^	0.59 ± 0.00 ^b^	<0.05	0.60 ± 0.00 ^B^	0.62 ± 0.01	0.63 ± 0.01	0.63 ± 0.01	ns	0.63 ± 0.01 ^A^	<0.001
**20:4 n-6**	3.20 ± 0.02 ^C^	3.31 ± 0.03 ^a,b^	3.39 ± 0.02 ^a^	3.29 ± 0.01 ^b^	<0.05	3.33 ± 0.02 ^B^	3.61 ± 0.02	3.72 ± 0.04	3.63 ± 0.03	ns	3.66 ± 0.02 ^A^	<0.001
**20:5 n-3**	2.20 ± 0.01 ^A^	2.15 ± 0.01	2.17 ± 0.01	2.15 ± 0.01	ns	2.15 ± 0.01 ^B^	2.17 ± 0.01	2.20 ± 0.01	2.18 ± 0.02	ns	2.19 ± 0.01 ^A^	<0.01
**22:5 n-3**	0.74 ± 0.01	0.72 ± 0.00	0.74 ± 0.01	0.73 ± 0.01	ns	0.73 ± 0.00	0.70 ± 0.00	0.72 ± 0.01	0.73 ± 0.01	ns	0.72 ± 0.01	ns
**22:6 n-3**	11.12 ± 0.05 ^C^	11.50 ± 0.13 ^b^	11.84 ± 0.07 ^a^	11.49 ± 0.05 ^b^	<0.05	11.31 ± 0.06 ^B^	12.30 ± 0.09	12.46 ± 0.11	12.26 ± 0.16	ns	12.34 ± 0.07 ^A^	<0.001
**PUFA**	34.33 ± 0.07 ^A^	33.88 ± 0.19	34.13 ± 0.09	33.89 ± 0.07	ns	33.97 ± 0.07 ^B^	34.10 ± 0.09	34.23 ± 0.15	34.25 ± 0.21	ns	34.19 ± 0.09 ^A,B^	<0.05
**DHA/EPA**	5.06 ± 0.01 ^C^	5.35 ± 0.03 ^b^	5.46 ± 0.01 ^a^	5.35 ± 0.03 ^b^	<0.05	5.39 ± 0.02 ^B^	5.66 ± 0.02	5.66 ± 0.03	5.61 ± 0.03	ns	5.64 ± 0.02 ^A^	<0.001
**ARA/EPA**	1.44 ± 0.00 ^C^	1.54 ± 0.01 ^a,b^	1.56 ± 0.01 ^a^	1.53 ± 0.01 ^b^	<0.05	1.54 ± 0.00 ^B^	1.66 ± 0.00	1.69 ± 0.01	1.66 ± 0.0	ns	1.67 ± 0.00 ^A^	<0.001

^A, B, C^ = LSMs with different capital letter superscripts between stages of development were significantly different (*p* < 0.01); ^a, b, c^ = LSMs with different lower-case superscripts between treatments were significantly different (*p* < 0.05).

## Data Availability

The nucleotide sequences of *myod* and *mrf4* gene fragments (accession numbers OM326825 and OM326824, respectively), generated in this study for Siberian sturgeon (*Acipender baerii*), were deposited in GenBank (NCBI).

## References

[1] Bronzi P., Rosenthal H. (2014). Present and Future Sturgeon and Caviar Production and Marketing: A Global Market Overview. J. Appl. Ichthyol..

[2] Bronzi P., Chebanov M., Michaels J.T., Wei Q., Rosenthal H., Gessner J. (2019). Sturgeon Meat and Caviar Production: Global Update 2017. J. Appl. Ichthyol..

[3] Ruban G.I. (2005). The Siberian Sturgeon Acipenser Baerii Brandt: Species Structure and Ecology.

[4] Ruban G.I., Birstein V.J., Waldman J.R., Bemis W.E. (1997). Species Structure, Contemporary Distribution and Status of the Siberian Sturgeon, *Acipenser baerii*. Sturgeon Biodiversity and Conservation.

[5] Gisbert E., Ruban G.I. (2003). Ontogenetic Behavior of Siberian Sturgeon, *Acipenser baerii*: A Synthesis between Laboratory Tests and Field Data. Environ. Biol. Fishes.

[6] Devitsina G.V., Kazhlayev A.A. (1993). Development of Chemosensory Organs in Siberian Sturgeon, *Acipenser baeri*, and Stellate Sturgeon, Acipenser Stellatus. J. Appl. Ichthyol..

[7] Gisbert E. (1999). Early Development and Allometric Growth Patterns in Siberian Sturgeon and Their Ecological Significance. J. Fish Biol..

[8] Näslund J., Johnsson J.I. (2016). Environmental Enrichment for Fish in Captive Environments: Effects of Physical Structures and Substrates. Fish Fish..

[9] Arechavala-Lopez P., Cabrera-Álvarez M.J., Maia C.M., Saraiva J.L. (2021). Environmental Enrichment in Fish Aquaculture: A Review of Fundamental and Practical Aspects. Rev. Aquac..

[10] McAdam S.O. (2011). Effects of Substrate Condition on Habitat Use and Survival by White Sturgeon (*Acipenser transmontanus*) Larvae and Potential Implications for Recruitment. Can. J. Fish. Aquat. Sci..

[11] Barton B.A., Iwama G.K. (1991). Physiological Changes in Fish from Stress in Aquaculture with Emphasis on the Response and Effects of Corticosteroids. Annu. Rev. Fish Dis..

[12] Peterson R.H., Martin-Robichaud D.J. (1995). Yolk Utilization by Atlantic Salmon (*Salmo salar* L.) Alevins in Response to Temperature and Substrate. Aquac. Eng..

[13] Gessner J., Kamerichs C.M., Kloas W., Wuertz S. (2009). Behavioural and Physiological Responses in Early Life Phases of Atlantic Sturgeon (*Acipenser oxyrinchus* Mitchill 1815) towards Different Substrates. J. Appl. Ichthyol..

[14] Baker D.W., McAdam D.S.O., Boucher M., Huynh K.T., Brauner C.J. (2014). Swimming Performance and Larval Quality Are Altered by Rearing Substrate at Early Life Phases in White Sturgeon, *Acipenser transmontanus* (Richardson, 1836). J. Appl. Ichthyol..

[15] Bates L.C., Boucher M.A., Shrimpton J.M. (2014). Effect of Temperature and Substrate on Whole Body Cortisol and Size of Larval White Sturgeon (*Acipenser transmontanus* Richardson, 1836). J. Appl. Ichthyol..

[16] Zubair S.N., Peake S.J., Hare J.F., Anderson W.G. (2012). The Effect of Temperature and Substrate on the Development of the Cortisol Stress Response in the Lake Sturgeon, *Acipenser fulvescens*, Rafinesque (1817). Environ. Biol. Fishes.

[17] Moriyama S., Ayson F.G., Kawauchi H. (2000). Growth Regulation by Insulin-like Growth Factor-I in Fish. Biosci. Biotechnol. Biochem..

[18] Fuentes E.N., Valdés J.A., Molina A., Björnsson B.T. (2013). Regulation of Skeletal Muscle Growth in Fish by the Growth Hormone—Insulin-like Growth Factor System. Gen. Comp. Endocrinol..

[19] Valente L.M.P., Moutou K.A., Conceição L.E.C., Engrola S., Fernandes J.M.O., Johnston I.A. (2013). What Determines Growth Potential and Juvenile Quality of Farmed Fish Species?. Rev. Aquac..

[20] Eissa N., Wang H.-P. (2016). Transcriptional Stress Responses to Environmental and Husbandry Stressors in Aquaculture Species. Rev. Aquac..

[21] Bischoff A.A., Kubitz M., Wranik C.M., Pfefferkorn H., Augustin C.B., Hagen W., Palm H.W. (2017). Fatty Acid Utilization of Pikeperch (*Sander lucioperca* (Linnaeus, 1758)) Larvae under Starvation Conditions during Early Development. Bull. Fish Biol..

[22] Tocher D.R. (2010). Fatty Acid Requirements in Ontogeny of Marine and Freshwater Fish. Aquac. Res..

[23] Aidos L., Valente L.M.P., Sousa V., Lanfranchi M., Domeneghini C., Giancamillo A.D. (2017). Effects of Different Rearing Temperatures on Muscle Development and Stress Response in the Early Larval Stages of *Acipenser baerii*. Eur. J. Histochem..

[24] Aidos L., Cafiso A., Bertotto D., Bazzocchi C., Radaelli G., Giancamillo A.D. (2020). How Different Rearing Temperatures Affect Growth and Stress Status of Siberian Sturgeon *Acipenser baerii* Larvae. J. Fish Biol..

[25] Aidos L., Vasconi M., Abbate F., Valente L.M.P., Lanfranchi M., Di Giancamillo A. (2019). Effects of Stocking Density on Reared Siberian Sturgeon (*Acipenser baerii*) Larval Growth, Muscle Development and Fatty Acids Composition in a Recirculating Aquaculture System. Aquac. Res..

[26] Food and Agriculture Organization of the United Nations (FAO) Fisheries and Aquaculture Department—Species Fact Sheets. www.fao.org.

[27] Folch J., Lees M., Sloane Stanley G.H. (1957). A Simple Method for the Isolation and Purification of Total Lipides from Animal Tissues. J. Biol. Chem..

[28] Christie W.W. (2003). Lipid Analysis.

[29] Vasconi M., Aidos L., Di Giancamillo A., Bellagamba F., Domeneghini C., Moretti V.M. (2019). Effect of Temperature on Fatty Acid Composition and Development of Unfed Siberian Sturgeon (*A. baerii*) Larvae. J. Appl. Ichthyol..

[30] Di Giancamillo A., Rossi R., Vitari F., Pastorelli G., Corino C., Domeneghini C. (2009). Dietary Conjugated Linoleic Acids Decrease Leptin in Porcine Adipose Tissue. J. Nutr..

[31] Aidos L., Cafiso A., Serra V., Vasconi M., Bertotto D., Bazzocchi C., Radaelli G., Di Giancamillo A. (2020). How Different Stocking Densities Affect Growth and Stress Status of *Acipenser baerii* Early Stage Larvae. Animals.

[32] Bustin S.A., Benes V., Garson J.A., Hellemans J., Huggett J., Kubista M., Mueller R., Nolan T., Pfaffl M.W., Shipley G.L. (2009). The MIQE Guidelines: Minimum Information for Publication of Quantitative Real-Time PCR Experiments. Clin. Chem..

[33] Livak K.J., Schmittgen T.D. (2001). Analysis of Relative Gene Expression Data Using Real-Time Quantitative PCR and the 2^−ΔΔCT^ Method. Methods.

[34] Gisbert E., Williot P., Castelló-Orvay F. (1999). Behavioural Modifications in the Early Life Stages of Siberian Sturgeon (*Acipenser baerii*, Brandt). J. Appl. Ichthyol..

[35] Gebauer T., Gebauer R., Císař P., Tran H.Q., Tomášek O., Podhorec P., Prokešová M., Rebl A., Stejskal V. (2021). The Effect of Different Feeding Applications on the Swimming Behaviour of Siberian Sturgeon: A Method for Improving Restocking Programmes. Biology.

[36] Rescan P.Y. (2001). Regulation and Functions of Myogenic Regulatory Factors in Lower Vertebrates. Comp. Biochem. Physiol. Part B Biochem. Mol. Biol..

[37] Wood A.W., Duan C., Bern H.A. (2005). Insulin-Like Growth Factor Signaling in Fish. International Review of Cytology.

[38] Patruno M., Sivieri S., Poltronieri C., Sacchetto R., Maccatrozzo L., Martinello T., Funkenstein B., Radaelli G. (2008). Real-Time Polymerase Chain Reaction, in Situ Hybridization and Immunohistochemical Localization of Insulin-like Growth Factor-I and Myostatin during Development of *Dicentrarchus labrax* (Pisces: Osteichthyes). Cell Tissue Res..

[39] Boucher M.A., McAdam S.O., Shrimpton J.M. (2014). The Effect of Temperature and Substrate on the Growth, Development and Survival of Larval White Sturgeon. Aquaculture.

[40] Boucher M.A., Baker D.W., Brauner C.J., Shrimpton J.M. (2018). The Effect of Substrate Rearing on Growth, Aerobic Scope and Physiology of Larval White Sturgeon *Acipenser transmontanus*. J. Fish Biol..

[41] Luo L., Ai L., Li T., Xue M., Wang J., Li W., Wu X., Liang X. (2015). The Impact of Dietary DHA/EPA Ratio on Spawning Performance, Egg and Offspring Quality in Siberian Sturgeon (*Acipenser baeri*). Aquaculture.

[42] Bell J.G., Ashton I., Secombes C.J., Weitzel B.R., Dick J.R., Sargent J.R. (1996). Dietary Lipid Affects Phospholipid Fatty Acid Compositions, Eicosanoid Production and Immune Function in Atlantic Salmon (*Salmo salar*). Prostaglandins Leukot Essent Fat. Acids.

[43] Lee S.-M. (2001). Review of the Lipid and Essential Fatty Acid Requirements of Rockfish (*Sebastes schlegeli*). Aquac. Res..

[44] Yanes-Roca C., Rhody N., Nystrom M., Main K.L. (2009). Effects of Fatty Acid Composition and Spawning Season Patterns on Egg Quality and Larval Survival in Common Snook (*Centropomus undecimalis*). Aquaculture.

[45] Ishizaki Y., Masuda R., Uematsu K., Shimizu K., Arimoto M., Takeuchi T. (2001). The Effect of Dietary Docosahexaenoic Acid on Schooling Behaviour and Brain Development in Larval Yellowtail. J. Fish Biol..

[46] Navarro J.C., Sargent J.R. (1992). Behavioural Differences in Starving Herring *Clupea harengus* L. Larvae Correlate with Body Levels of Essential Fatty Acids. J. Fish Biol..

[47] Hauville M.R., Main K.L., Migaud H., Gordon Bell J. (2016). Fatty Acid Utilization during the Early Larval Stages of Florida Pompano (*Trachinotus carolinus*) and Common Snook (*Centropomus undecimalis*). Aquac. Res..

[48] Rowlerson A., Veggetti A. (2001). Cellular Mechanisms of Post-Embryonic Muscle Growth in Aquaculture Species. Muscle Dev. Growth.

[49] Alami-Durante H. (1990). Growth of Organs and Tissues in Carp (*Cyprinus carpio* L.) Larvae. Growth Dev. Aging.

[50] Galloway T.F., Kjorsvik E., Kryvi H. (1999). Muscle Growth in Yolk-Sac Larvae of the Atlantic Halibut as Influenced by Temperature in the Egg and Yolk-Sac Stage. J. Fish Biol..

[51] Nathanailides C., Stickland N.C., Lopez-Albors O. (1995). Influence of Prehatch Temperature on the Development of Muscle Cellularity in Posthatch Atlantic Salmon (*Salmo salar*). Can. J. Fish. Aquat. Sci..

[52] Steinbacher P., Haslett J.R., Sänger A.M., Stoiber W. (2006). Evolution of Myogenesis in Fish: A Sturgeon View of the Mechanisms of Muscle Development. Anat. Embryol..

[53] Zhang Z., Fu Y., Guo H., Zhang X. (2021). Effect of Environmental Enrichment on the Stress Response of Juvenile Black Rockfish *Sebastes schlegelii*. Aquaculture.

[54] Murtaza M.u.H., Zuberi A., Ahmad M., Amir I., Kamran M., Ahmad M. (2020). Influence of Early Rearing Environment on Water-Borne Cortisol and Expression of Stress-Related Genes in Grass Carp (*Ctenopharyngodon idella*). Mol. Biol. Rep..

[55] Maradonna F., Bavestrello G., Cardinali M., Olivotto I., Cerrano C., Giovine M., Carnevali O. (2003). Role of Substrate on Larval Development of the Freshwater Teleost *Pelvicachromis pulcher*. Mol. Reprod. Dev..

[56] Johnston I.A. (2006). Environment and Plasticity of Myogenesis in Teleost Fish. J. Exp. Biol..

